# Effective Model Update for Adaptive Classification of Text Streams in a Distributed Learning Environment

**DOI:** 10.3390/s22239298

**Published:** 2022-11-29

**Authors:** Min-Seon Kim, Bo-Young Lim, Kisung Lee, Hyuk-Yoon Kwon

**Affiliations:** 1 Department of Industrial Engineering, Seoul National University of Science and Technology, 232 Gongneung-ro, Nowon-gu, Seoul 01811, Republic of Korea; 2 Division of Computer Science and Engineering, Louisiana State University, Baton Rouge, LA 70803, USA; 3 Department of Industrial Engineering, The Research Center for Electrical and Information Technology, Seoul National University of Science and Technology, 232 Gongneung-ro, Nowon-gu, Seoul 01811, Republic of Korea

**Keywords:** event classification, text streams, distributed learning, continual learning, dynamic model update

## Abstract

In this study, we propose dynamic model update methods for the adaptive classification model of text streams in a distributed learning environment. In particular, we present two model update strategies: (1) the entire model update and (2) the partial model update. The former aims to maximize the model accuracy by periodically rebuilding the model based on the accumulated datasets including recent datasets. Its learning time incrementally increases as the datasets increase, but we alleviate the learning overhead by the distributed learning of the model. The latter fine-tunes the model only with a limited number of recent datasets, noting that the data streams are dependent on a recent event. Therefore, it accelerates the learning speed while maintaining a certain level of accuracy. To verify the proposed update strategies, we extensively apply them to not only fully trainable language models based on CNN, RNN, and Bi-LSTM, but also a pre-trained embedding model based on BERT. Through extensive experiments using two real tweet streaming datasets, we show that the entire model update improves the classification accuracy of the pre-trained offline model; the partial model update also improves it, which shows comparable accuracy with the entire model update, while significantly increasing the learning speed. We also validate the scalability of the proposed distributed learning architecture by showing that the model learning and inference time decrease as the number of worker nodes increases.

## 1. Introduction

Twitter is one of the popular social networking services dealing with text streams, which provides a fast and interactive channel where the users write tweets and obtain access to the written tweets related to the latest events [1]. There have been lots of research efforts to classify the tweets and detect certain types of events using the collected tweets, focusing on the offline classification model [2,3]. Because tweets have short texts, it causes great difficulties in classification. Batool et al. [2] extracted knowledge from tweets and classified tweets based on the semantics of tweets. Shin et al. [3] proposed a text classification model for detecting cyber-security-related tweets by introducing two contrastive word embedding models that are positive and negative to the target events.

Considering that new information and responses to events are rapidly generating, it is important to quickly reflect data generated in real time into the model. However, applying the large-scale data streams to the classification model dynamically causes many limitations because it requires high costs in re-training the model to reflect the new data generated in real time. To confirm the overhead of the learning process, we conduct a preliminary experiment that compares the data ingestion, learning, and inference time by scaling the number of tweets. For this experiment, we used one of the datasets used in the experiments, i.e., the CSI dataset, and the bidirectional LSTM (Bi-LSTM)-based classification model. Table 1 shows the results that clearly confirm the learning process is a bottleneck in the entire classification pipeline considering its portion and the increased ratio according to the data scale compared to the ingestion or inference time. In the previous study [4], the relatively high portion of learning time over the data ingestion time has been presented to show the necessity of effective data ingestion to retain the ingested datasets for further learning. In this study, we focus on how the portion of the learning time becomes longer compared to the ingestion time as the data increases, showing the necessity of the efficient model update for large-scale data streams. Specifically, the learning time over the ingestion time is 5.43 times in the case of 200,000 tweets, whereas it is 4.42 times in the case of 50,000 tweets. This indicates that we need an effective model update method in an environment where the streaming data continuously flows in by efficiently learning the classification model while maintaining the model accuracy.

In this study, we deal with the problem of updating the text classification models dynamically in a distributed environment to respond to the streaming data flowing in at a fast speed. In this regard, we claim two research objectives of this study. First, according to the continuously changing event trends in the streaming data, we need to efficiently reflect the changes into the existing model to respond to those changes in a real-time manner. Second, considering that large-scale streaming data flows at a high speed, we need to design a scalable architecture based on the distributed environment, accelerating the learning and inference speed of the model. We note that the existing classification models for the streaming data were not effective in terms of their efficiency and scalability in reflecting the dynamic changes into the model in a distributed environment.

There have been lots of research efforts to increase the performance of event classification in data streams [3,5,6,7,8,9,10,11,12]. Because the performance of the classifier is significantly affected by the underlying embedding models, effective word-embedding methods have been proposed [3,6,11]. On the other hand, a few studies have investigated building a set of classifiers and selectively utilizing them to respond to non-stationary data streams [9,10,12]. However, a scalable architecture for the streaming event classification in a distributed environment, which is required to efficiently respond to detect and track the events from data sources with large volumes and high velocity, has not been considered before.

Learning the model in a centralized server has an inherent limitation in providing scalability for dealing with massive-scale data. Therefore, distributed learning has evolved as the solution by distributing the overhead of maintaining a global model in a centralized server into multiple nodes. Lots of recent research efforts have focused on the cooperation of multiple nodes to update a global model [13,14,15,16,17]. On the other hand, the efficient reflecting of local changes in each local model in a distributed environment has also been addressed in several studies [18,19,20,21]. However, there have been no research efforts to dynamically update the distributed classification model to respond to the changing non-stationary event streams.

To respond to non-stationary data streams, distributed online learning has been explored in various fields [22,23,24,25,26], and they target common research goals of dynamically updating the model in a distributed environment. All previous studies focused on improving the model performance targeting a specific model, but it is essential to immediately update the model by reflecting newly generated data streams to the model in order to respond to a new event trend in real-time data streams. In this regard, our distinguishing research goal that is different from the existing distributed online learning methods is proposing an efficient model update method while minimizing the accuracy loss, not improving the model performance. Furthermore, we do not focus on a specific classification model and, instead, identify common trainable modules for the typical classification models and apply the update strategies to the various classification models.

In this study, we propose the dynamic model update methods for the adaptive classification model of data streams in a distributed learning environment. Based on the scalable architecture in a distributed environment, we present two model update strategies: (1) the entire model update and (2) the partial model update. The former aims to maximize the model accuracy by accumulating all the datasets including recent datasets and rebuilding the model periodically as the trends in data streams change. Accordingly, its accuracy increases over time as the data increases, but the learning time significantly increases at the same time. The latter fine-tunes the model only with a limited number of recent datasets, noting that the data streams are dependent on a recent event. Therefore, it accelerates the learning speed while maintaining a certain level of accuracy. To verify the effectiveness of the proposed update strategies, we extensively evaluate not only fully trainable language models based on CNN, RNN, and Bi-LSTM but also a pre-trained word-embedding model based on BERT. In particular, we identify the partial modules that can be effectively updated with a marginal overhead for all the models in common.

The contributions of the paper can be summarized as follows:

- We design a scalable classification model based on a distributed learning environment that enhances the parallelism of the model learning. Therefore, it can resolve the bottleneck that occurred during the learning process in the entire event stream classification pipeline (Section 3.1).
- Based on a distributed learning architecture, we propose two kinds of model update strategies: (1) the entire model update and (2) the partial model update. Because they have their distinguishing properties in terms of learning efficiency and model accuracy, they can be selectively chosen according to the needs of the target applications (Section 3.3 and Section 3.4).
- As the target classification models, we consider not only fully trainable language models based on CNN, RNN, and Bi-LSTM but also a pre-trained word-embedding model based on BERT. In particular, we identify the trainable partial modules that are commonly applied in the deep learning-based classification models (Section 3.2).
- We conduct extensive experiments using two real tweet datasets and show the effectiveness of the proposed update strategies. Specifically, the entire model update gradually improves the classification accuracy in the range of 28.96∼58.63% compared to the pre-trained offline model; the partial model update improves it in the range of 12.34∼50.92%, while significantly reducing the learning time by 69.35∼93.95% compared to entire model update strategy. We also confirm the scalability of the proposed distributed learning architecture by showing that compared to using a single worker node, the learning time decreases by 34.03% in the entire model update and by 45.21% in the partial model update, respectively, when using three worker nodes (Section 4).

The remainder of this paper is organized as follows. In Section 2, we describe the related work. In Section 3, we present the proposed dynamic model update methods. In Section 4, we present the experimental results. In Section 5, we conclude the paper and discuss future work.

## 2. Related Work

### 2.1. Data Stream Classification

Lots of research efforts for detecting the events based on the classification model from the streaming data have been conducted. Mittal et al. [27] described the necessity of incremental algorithms to achieve the consistent accuracy of the classifier in streaming environments to respond to the concept drift. They evaluated five data stream mining algorithms in various drifting settings and found that none of each algorithm outperforms the others in all settings due to a trade-off between the model accuracy and training speed of the algorithms. Nishida et al. [5] proposed a classification model for streaming tweets by examining the changes in class distributions and probabilities of word occurrences. Weiler et al. [6] monitored shifts in the inverse document frequency (IDF) of terms to identify events from large-scale SNS streams. Zyblewski et al. [9] proposed a dynamic classifier based on ensemble selection methods to classify the non-stationary data streams. Shin et al. [3] proposed a text classification model that detects cyber-security-related tweets by introducing a contrastive word-embedding model that defines positive and negative embedding models to the target event. Malialis et al. [12] proposed a new density-based active learning strategy based on the similarity in the latent space for non-stationary and imbalanced data streams. Nguyen et al. [7] extracted and tracked social events on real-time data streams by aggregating discrete signals representing relevant keywords from the tweets collected by the event categorization. Eddaoudy et al. [28] proposed a distributed machine learning model on streaming data based on Apache Spark to learn the event streams and to predict events in real time.

The previous studies have not considered the classification model that can be dynamically updated as the input stream changes. In this problem, because event trends fluctuate over time, we need to periodically re-train the classification model to maintain the model accuracy. However, re-training of the entire model iteratively requires considerable costs and time. To resolve this challenge, we propose a partial model update strategy that effectively updates the model with a marginal update overhead while maintaining the model accuracy.

### 2.2. Distributed Learning

Distributed learning has become popular due to the explosion in the size and complexity of datasets to be learned. Gupta et al. [29] proposed a model partitioning strategy over multiple agents and further incorporated it with semi-supervised learning using a few labeled samples. Huang et al. [30] combined approximate augmented Lagrangian function with time-varying gaussian noise addition in a distributed learning framework with differential privacy, providing performance improvement. Chen et al. [15] updated a central model in an asynchronous manner to cope with the heterogeneity of distributed edge devices. Wang et al. [16] proposed a distributed modulation classification model based on the cooperation of multiple edge devices and a model averaging algorithm. They achieved lower computing overhead than centralized modulation classification to achieve a similar convergence speed. Park et al. [20] presented a communication-efficient distributed learning framework that enables edge nodes to proactively and independently react to local changes. Gao et al. [31] divided the learning process of graph neural networks into two stages to resolve the mismatch between the graphs. They first learned nonlinear representations from raw data at the training stage and retrained the linear representations at the testing stage.

Recently, Apache Spark [32] became a popular choice to convey big data analytics or machine learning tasks for large-scale datasets based in a distributed environment. Several research efforts for distributed learning of neural networks have been conducted based on Apache Spark. Dunner et al. [33] proposed practical techniques to achieve the best performance in Apache Spark, targeting any distributed algorithms and infrastructures. Zhao et al. [34] proposed a scalable stochastic optimization method on Apache Spark that achieves both computation and communication efficiency. Alkhoury et al. [35] proposed the communication-efficient distributed learning model on Apache Spark and applied it to image segmentation on large-scale datasets. In this study, we deal with the dynamic model update problems on deep learning-based classification models on Apache Spark, which has not yet been studied before.

### 2.3. Continual Learning

Continual learning deals with the problem of learning from potentially infinite data streams with the goal of preserving and extending acquired knowledge. This is also referred to as lifelong learning or incremental learning in the literature. The main challenge with continual learning is catastrophic forgetting, which is a tendency of neural networks that forget previously learned knowledge in the learning process for new data. Various methodologies [36,37,38,39,40,41,42,43,44,45,46,47,48,49,50] for continual learning have been proposed to effectively handle sequential new tasks while maintaining the classification accuracy of previous tasks. The methodologies can be largely categorized into four groups: (1) regularization-based methods, (2) knowledge distillation methods, (3) rehearsal-based methods, and (4) dynamic architecture methods.

Regularization-based methods modify the gradient of parameters for optimization by assigning constraints to the weights to be updated. Kirkpatrick et al. [36] proposed a regularization to model parameters by selectively learning important weights for old tasks. In a similar way, Zenke et al. [37] extended the loss function to penalize changes to parameters that are unimportant for old tasks. Mirzadeh et al. [38] assessed the impact of different training regimes on catastrophic forgetting and widened the curvature of each task to prevent catastrophic forgetting. Yoon et al. [51] decomposed the entire model parameters into task-generic parameters and task-specific parameters to maintain inference accuracy between different tasks.

Knowledge distillation methods attempt to alleviate the catastrophic forgetting issue by distilling the knowledge learned from the previous data when learning new tasks. Li et al. [39] preserved the knowledge learned from past tasks by using a distillation loss. Rebuffi et al. [40] re-defined the loss function using both classification loss and distillation loss to distill the knowledge learned from the existing tasks when learning new tasks. Castro et al. [41] trained the deep learning-based models by minimizing both cross-entropy loss to learn new classes and distillation loss to retain the previous knowledge.

Rehearsal-based methods build and store a memory of the knowledge learned from old tasks and periodically replay the model to strengthen connections with previous knowledge. Rebuffi et al. [40] maintained the exemplar set of previous representative data samples in memory and updated them when new data are observed. Chaudhry et al. [42] built a dynamic episodic memory of parameter gradients during the learning process for leading to a faster learning process while not forgetting each individual task. Wang et al. [43] performed meta-learning of the model to learn a better initialization for local adaptation. Some studies generated synthetic data by learning the data distribution of previous tasks and used them in learning new tasks. Shin et al. [44] proposed a novel framework with a deep generative model to enable previous training data to be sampled and interleaved with those for a new task. Wang et al. [45] replayed data sampled from the conditional generative adversarial network. They selectively stabilized the parameters of the discriminator for discriminating the pairs of old unlabeled data and their predicted pseudo-labels to overcome the catastrophic forgetting of unlabeled data.

Dynamic architecture methods are typically used in task-incremental learning to learn the task-specific parameters or networks. Rebuffi et al. [49] introduced universal parametric families of neural networks that contain both domain-shared parameters among multiple domains and domain-specific modular adapters and attached them to the network for new tasks. Rusu et al. [46] proposed a training method that grows a network hierarchically to handle new coming data. Mallya et al. [47] pruned and retrained the network by obtaining the sparsity masks for the tasks and utilizing them to freeze the corresponding network weights. Mallya et al. [48] masked unimportant parameters for previous tasks to train the parameters for new tasks. Ashfahani et al. [50] proposed an autonomous deep learning algorithm based on the self-constructing structure generating different depths and widths. Cano et al. [52] proposed an ensemble architecture with the concept drift to deal with imbalanced data streams by reflecting them to the model in an online manner.

Previous studies for continual learning focused on preserving and extending the acquired knowledge in the process of learning new data to prevent catastrophic forgetting. In contrast, in this study, we focus on the efficient model update that can respond to the continuously changing data streams while maintaining a certain level of classification accuracy.

### 2.4. Distributed Online Learning

Time-dependent data learning has drawn a lot of attention to an online learning framework in the last few years. Specifically, introducing a distributed environment aims to minimize the computing overhead and to provide scalability to the increased data scales while maintaining the model performance. Tekin et al. [22] proposed distributed online learning algorithms that lead each processor to learn itself for maximizing the total expected rewards from its own actions without the interaction between processors. Zhang et al. [23] explored an online conditional gradient algorithm with simple linear optimization steps in the distributed online learning setting. Li et al. [24] developed a privacy-preserving distributed online learning framework by building independent local models based on local datasets and exchanging intermediate parameters with neighboring nodes to achieve convergence. They showed that the Euclidean distance of all learnable parameters became shorter over iterations. Wang et al. [14] managed the dynamic resource constraints by adaptively and periodically choosing the optimal global aggregation frequency, considering the network cost and model performance simultaneously. Wu et al. [26] proposed a distributed hierarchical online learning approach to enhance the robustness by reducing the long-term cost when converging to a new local optimal.

In this study, we target the problem where the trends in the data streams are continuously changing. In this problem, we need to update the model periodically to reflect new data streams according to the changing events, requiring intensive learning overhead. To address the problem effectively, we propose a distributed architecture on Apache Spark that can reflect real-time data streams by updating the model with an appropriate time overhead. The architecture provides methods for dynamically updating the model in a distributed learning environment by considering both learning efficiency and classification accuracy.

### 2.5. Summary

In this study, we aim to provide an efficient model update method in a distributed environment while maintaining a certain level of accuracy. Table 2 summarizes the coverage of related studies in terms of (1) streaming classification, (2) distributed learning, (3) dynamic model update, and (4) model learning efficiency, which are the four main focuses of this study. As shown in the table, none of the previous studies have considered all four focuses of this study. Distributed online learning, which was described in Section 2.4, has dealt with the most similar issues to our study but has not considered an environment where large amounts of data flow in, requiring the immediate reflection of them to the model [14,23]. As a result, they are not appropriate to be used for updating the classification model efficiently to respond to the streaming data flowing in at a fast speed. To the best of our knowledge, our study is the first research effort to update the model in an online manner to classify the streaming data in a distributed environment.

A preliminary version of this study was presented as a conference short paper [4]. In this paper, we fully rewrite and extensively extend it. The major extensions include (1) proposing a completely new dynamic model update strategy, partial model update, which is a more effective and practical method by providing immediate model updates while maintaining a certain level of accuracy, compared to the entire model update strategy in the preliminary version, (2) extensive experiments on two tweet datasets using two model update strategies, (3) extensive applications of the idea to not only the fully trained language models based on CNN, RNN, and Bi-LSTM but also the pre-trained word-embedding model based on BERT, and (4) the detailed and extensive literature reviews for related work.

## 3. Proposed Method

### 3.1. Overall Framework

**Figure 1:**
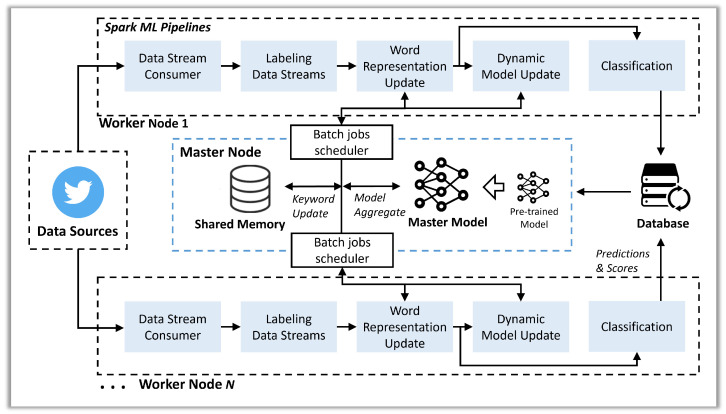
Overall framework architecture.

Figure 1 shows the overall architecture of our proposed framework. For distributed learning and classification, our framework is designed to run on an Apache Spark cluster with one master node and a set of worker nodes. We run Spark ML pipelines on the cluster to classify streaming data in real time and update classification models for each time window in a distributed manner. In our experimental settings, we prepare a total dataset related to the target event in advance and feed them by the time window to control the data streams according to our intention. Here, we use a set of seed keywords defined for an event. Our classification model on each worker node can determine if each streaming data (e.g., tweet selected using the seed keywords) is actually related to the event in real time. To efficiently handle large-scale streaming data in a scalable manner, we distribute the collected data to the worker nodes, which take data using the data stream consumer. We develop our distributed training and classification pipelines using the transformers (e.g., feature vector generation, classification using a learned model) and estimators (i.e., learning algorithms) provided by Spark’s machine learning library (Apache Spark MLlib). The word representation update module processes each data to use it as an input for model training or classification and has two main processing steps: (1) pre-processing step, including stemming and lemmatization, and (2) word-embedding step, including text vectorization. To dynamically update our classification model using recently collected data, our framework has the batch jobs scheduler that updates the frequent word list and coordinates model updates. For the model updates, our framework assumes that all or a part of the newly collected data in the current window are labeled by using any available labeling techniques, such as crowdsourcing-based labeling and emerging approaches (e.g., active learning, semi-supervised learning, and self-supervised learning) [53,54,55,56,57,58]. It is worth noting that our study focuses on reflecting non-stationary features presented in real-time data streams more efficiently on the model, not improving model performance using more accurate labeling. For distributed model updates on the cluster, we initialize a deep learning-based model on the driver of the master node and ship its serialized version to the worker nodes with model parameters. Each worker node deserializes the model, trains it using its chunk of data, and sends its gradients back to the master node, which aggregates the gradients and updates the master model. We distribute the updated master model to worker nodes and replace the classification model with the updated one to classify streaming data in real time.

### 3.2. Classification Model

In the data streams, the trends of the data can be dynamically changed over time. In particular, the streaming data are significantly sensitive to recently occurred events. Therefore, we need to respond to them by incrementally updating the model using the newly collected data, in particular, focusing on recent data. However, as streaming data are continuously reached, reflecting them to the existing models is a challenging issue because the learning overhead of the models significantly increases as described in Table 1. In this study, we focus on the efficient model update reflecting the evolving recent trends in the streaming data while maintaining the accuracy of the classification model. In particular, we consider the learning model in a distributed environment to deal with massive-scale datasets with continuously increasing volumes. We present two kinds of model update strategies: (1) the entire model update (described in Section 3.3) and (2) the partial model update (described in Section 3.4).

**Figure 2:**
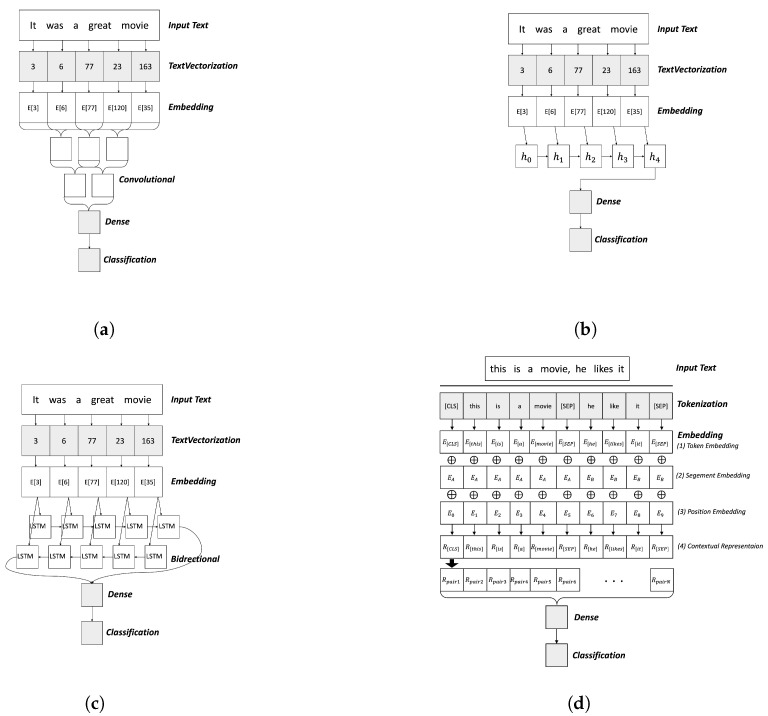
Deep learning-based architectures with trainable layers (**a**) CNN, (**b**) RNN, (**c**) Bi-LSTM, (**d**) BERT.

For dynamic model updates in this study, we employ learning architectures based on three typical neural networks (CNN, RNN, and Bi-LSTM) and a transformer-based pre-trained language model (BERT) as shown in Figure 2 in which trainable layers are highlighted in gray. In the CNN-, RNN-, and Bi-LSTM-based architectures, an embedding layer consists of a 100-word sequence of each sentence. A sequence of integers with text data is fed as input, which corresponds to word indices in a sentence to the layer. The layer generates up to 100-word embeddings for each text using the top-5000 frequently occurred keywords in the text corpus and learns the vector representation of each word. We denote this embedding model as *event-specific word embedding*, fully training the embedding layer with the datasets for the target event, and we also apply it to the BERT-based architecture to compare it with the original BERT pre-trained word embedding. By continuously updating the top 5000 keywords, the embedding layer can reflect trend changes in streaming data.

In the CNN-based architecture (Figure 2a), the word embeddings of input data are connected to a 1D convolutional layer that extracts features by sliding along the word embeddings in sequence to look at embeddings of multiple consecutive words at the same time.In the RNN-based architecture (Figure 2b), the ordered word embeddings of data (i.e., an embedding sequence) are used as an input to a hidden layer that processes the sequence in the forward direction. In the Bi-LSTM-based architecture (Figure 2c), by processing the embedding sequence in the forward and backward directions, we can keep track of information in the sequence from both directions. Each architecture has a dense layer with the sigmoid activation function as its last layer to perform binary classification for each data. The BERT-based architecture is described in Section 3.5.

### 3.3. Entire Model Update

**Figure 3:**
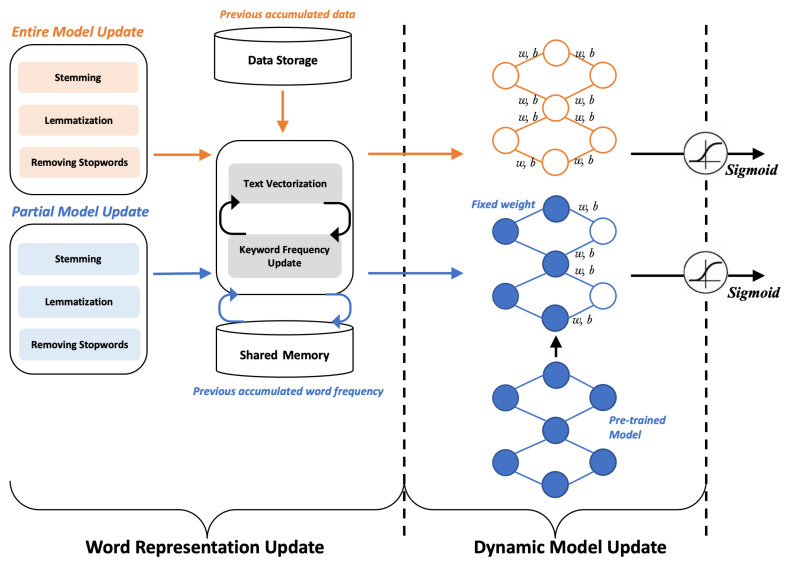
Continual machine learning pipeline for classification supporting dynamic model update.

As the first strategy for dynamic model updates, we propose the *entire model update*, which trains a new classification model from scratch using all accumulated data in each time window, as shown in Figure 3. It is worth noting that the word frequency list is being continuously updated as our framework takes new data, and we utilize the updated top-5000 frequent keywords in each time window for the entire model update to reflect the recent trends of streaming data. After the model is trained with new input vectors, it is serialized and shipped to the distributed worker nodes and used to classify the newly generated data streams during the upcoming time window. Even though the entire model update can generate a more accurate model by digesting all accumulated data, one main limitation of this strategy is limited scalability, because we need to keep all collected data on our Spark cluster, and consequently, the model learning time will continuously increase as we use more data for training in each window, as evidenced in Table 1.

### 3.4. Partial Model Update

To address the limited scalability of the entire model update strategy, we propose a lightweight strategy, called the *partial model update*, that updates only a portion of the classification model based on the pre-trained offline model, as shown in Figure 3. In this strategy, we pre-train a classification model using previously collected data in an offline manner and fine-tune only a part of the model using newly streamed data in the current time window. Specifically, only the dense layer is fine-tuned with the newly streamed data in each time window. Like the entire model update strategy, the word frequency list is continuously updated as our framework takes new data, and we utilize the updated top-5000 frequent keywords in each time window to reflect the recent trends of streaming data. Unlike the entire model update strategy, the partial model update strategy does not keep all accumulated data on a cluster because it needs only newly streamed data in the current window for fine-tuning. Assuming a similar number of streaming data in each window, we expect that the partial model update (i.e., fine-tuning ) would require a consistent learning time for all windows, tackling the scalability issue of the entire model update strategy.

### 3.5. Application to Pre-Trained Embedding Model

In this study, we also consider pre-trained embedding models, which have been known to show better performance than the typical fully trained deep learning models in most natural language processing (NLP) tasks. Devlin et al. [59] proposed Bidirectional Encoder Representations from Transformers (BERT) that is trained using unlabeled data extracted from BooksCorpus [60] and English Wikipedia. It can be fine-tuned by adding the output layer tailored to target NLP tasks. There is a major computational advantage of pre-computing representations of input data and then using lightweight models on top of these representations to apply them to downstream tasks. As shown in Figure 2d, BERT reads a complete sequence of words in parallel, enabling the model to understand each word’s context as a result of the relationship with neighboring words. We update the top-5000 frequent keyword sets in the tokenization process similar to the other models before embedding processes (e.g., token embedding, segment embedding, and position embedding). Here, we set the maximum word sequences for each sentence as 100. By embedding updated keywords that are considered important at each time window, this model can also adaptively respond to the changes in input data streams. BERT has a total of 110 million trainable parameters, and such high model complexity calls for expensive computational resources and extremely excessive training costs. Thus, the iterative re-training of BERT to respond to the streaming events is not a feasible approach [61,62]. Therefore, we consider only the partial model update strategy for the BERT-based architecture in our proposed framework.

## 4. Performance Evaluation

In this study, we perform extensive experiments to verify the effectiveness of our proposed framework using real-world datasets. The experiments aim to show that our strategies for the dynamic model updates are feasible to respond to the trend changes in the data streams in terms of both model accuracy and learning efficiency. Here, we apply two model update strategies (entire and partial) described in Section 3. We collected two kinds of datasets related to different target events from Twitter: (1) the cybersecurity intelligence (CSI) dataset and (2) the disaster dataset. To control the input data events as the time varies as we want, we prepare two kinds of datasets for each event: (1) the relevant dataset to the event and (2) the irrelevant dataset to the event. Then, we divide them into sub-datasets to feed each sub-dataset to each time window. The datasets used for training the model in each time window depend on each update strategy. We extensively apply our proposed dynamic model update strategies into three typical neural networks (CNN, RNN, and Bi-LSTM) and a transformer-based pre-trained model (BERT). We measure the classification accuracy and the learning time as the evaluation metrics.

### 4.1. Datasets

Table 3 shows the dataset name, the number of tweets, the number of time windows, and the time period for our datasets. By leveraging the accounts and keyword sets that are relevant to the target event, we collect the actual tweets by crawling them in time order. We use 80 percent of the data in each time window to train and validate the model and the remaining 20 percent to test the model performance. We explain the details of CSI and disaster datasets in Section 4.1.1 and Section 4.1.2, respectively.

#### 4.1.1. CSI Dataset

For collecting the CSI-related dataset, we focus on the tweets containing the keyword ‘exploit’. Through the analysis engine on the target tweets by Recorded Future (https://www.recordedfuture.com (accessed on 31 October 2022)), we obtain 100 accounts and 639 keywords relevant to the cyber-security, e.g., ‘Internet-security’, ‘flaw’, ‘PoC’, and ‘CVE’. We collect all the tweets from 2007 to 2015 posted by the selected accounts. Then, we filter only the tweets containing at least one keyword in the relevant keyword set and define them as the CSI-related dataset. For the CSI-unrelated dataset, we collect random tweets containing general keywords, which are in the top-10 most commonly used English words, such as ‘the’, ‘to’, and ‘a’, based on an analysis of the Oxford English Corpus (http://oxforddictionaries.com/us/words/the-oxford-english-corpus (accessed on 31 October 2022)). This dataset has one million tweets including 500,000 CSI-related tweets and 500,000 CSI-unrelated tweets. Examples of CSI-related tweets are as follows: “A very deep dive into iOS Exploit chains found in the wild” and “Binary Exploitation—Buffer Overflow Explained in Detail”. We use 400,000 tweets to pre-train the model and an additional 100,000 tweets to re-train the model for each time window, with the same portion between CSI-related and CSI-unrelated datasets.

#### 4.1.2. Disaster Dataset

For the disaster-related dataset, we use a disaster-related keyword set consisting of 24 keywords (e.g., Flood, Epidemics, Windstorm) identified by Apronti et al. [63]. Since some of them could not be related to actual disasters, we collect the tweets satisfying the following conditions: (1) containing at least two keywords in the disaster keyword set, (2) having more than 10 characters, and (3) having more than five distinct words. We collect tweets satisfying the conditions posted from 2007 to 2015. For the disaster-unrelated data, we collect them in the same way as the CSI-unrelated dataset. This dataset has 1.4 million tweets including 700,000 disaster-related tweets and 700,000 disaster-unrelated tweets. Examples of disaster-related tweets are as follows: “Ian Downgraded to Tropical Storm, Flooding Threats Remain.” and “typhoon linpha causes flooding in northern philippines storm natural disaster”. We use 400,000 tweets to pre-train the model and an additional 200,000 tweets for each time window, with the same portion between disaster-related and disaster-unrelated datasets.

### 4.2. Experimental Methods and Environments

For the experiments, we use one master node and three worker nodes with Apache Spark 2.4.7 managed by Hadoop Yarn. Each node is equipped with Intel Xeon Silver 4210R 2.40 GHz CPU and 32 GB RAM and runs Ubuntu 18.04. To focus on the distributed environments, we only utilize the CPU-based environments without GPU devices. To apply the proposed method to various deep learning models, we evaluate four kinds of deep learning-based classification models: (1) CNN, (2) RNN, (3) Bi-LSTM, and (4) BERT. For training the event-specific word embedding of the first three models, we feed a sequence of integers with 400,000 data samples as input and learn the vector representation of each word.

For the model based on CNN, we employed a one-dimensional convolutional neural network layer with 256 units for feature extraction and one hidden layer for the classifier. For the model based on RNN, we adapted three SimpleRNN layers with 256 units for feature extraction and one hidden layer for the classifier. For the model based on Bi-LSTM, we adapted the Bi-LSTM layer with 128 units for feature extraction and one hidden layer after the embedding layer for the classifier. For all models, we adapted the dense layer with the sigmoid activation function after the hidden layer and performed binary classification for input tweets using the Adam optimizer. We commonly set the number of epochs to 10, the batch size to 128, and binary cross-entropy as a loss function in all settings. Using Elephas Estimator (https://github.com/maxpumperla/elephas (accessed on 31 October 2022)) supported by Spark MLlib, we run distributed classification models at scale on Apache Spark. For the fine-tuning of BERT-based architecture, we use another BERT model supported by SparkNLP (https://github.com/JohnSnowLabs/spark-nlp (accessed on 31 October 2022)) because we actually observe that the fine-tuning of BERT-based architecture only for the final dense layer using Elephas Estimator takes more than an hour in processing one epoch with 100,000 data samples, which is infeasible to apply to the dynamic model update in the streaming classification. SparkNLP utilizes the transformer itself of Spark MLlib without the process of abstracting the learning algorithm for customizing the transformer tailored to the datasets, efficiently fine-tuning the model. Although there might occur accuracy loss in the trained model, we use the SparkNLP-based approach to focus on the efficient model update according to our research goal.

### 4.3. Experimental Results

#### 4.3.1. Model Accuracy

**Figure 4:**
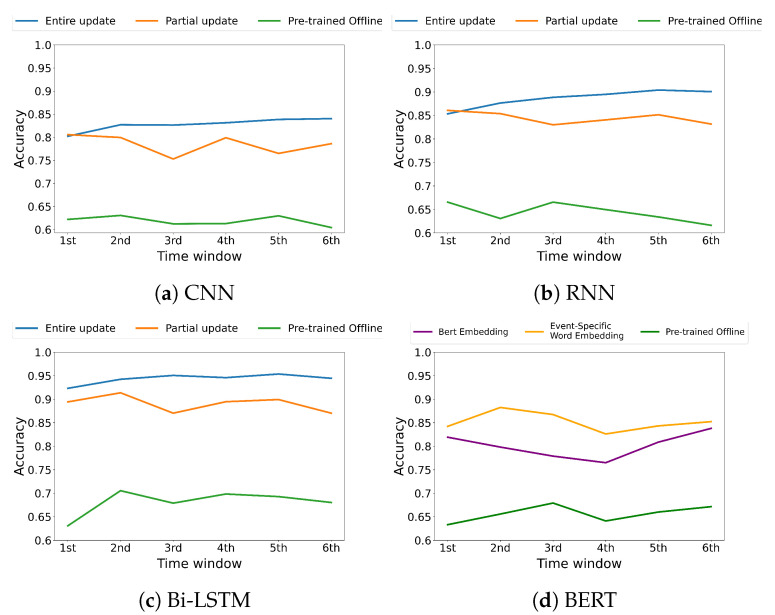
Accuracy comparison between (1) Pre-trained offline, (2) Entire model update, and (3) Partial model update on CSI dataset.

**Figure 5:**
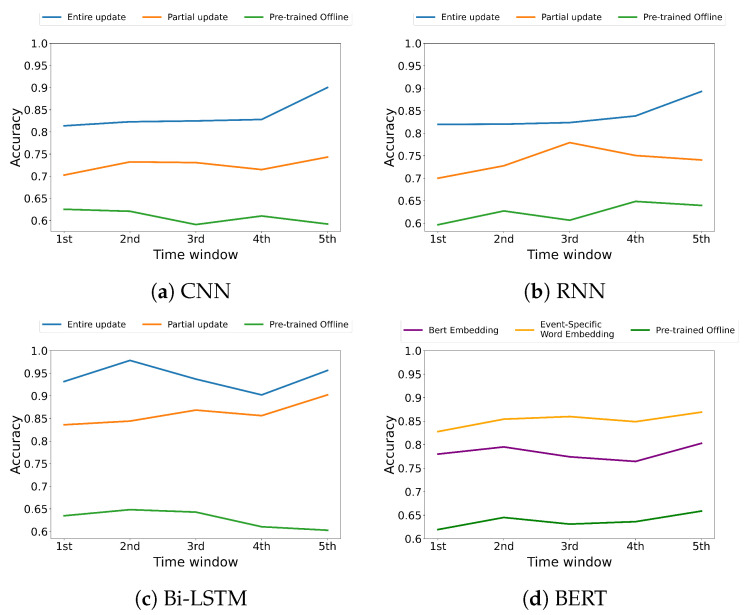
Accuracy comparison between (1) Pre-trained offline, (2) Entire model update, and (3) Partial model update on disaster dataset.

Figure 4 and Figure 5 represent the accuracy over time when each strategy (i.e., (1) Pre-trained offline, (2) Entire model update, (3) Partial model update) has been adopted on the CSI and disaster datasets, respectively. We note that both entire and partial update strategies show a distinct accuracy improvement compared to the strategy where the initial pre-trained offline model has been utilized for the inference. The results show that the entire model update improves the classification accuracy by 28.96∼58.63% compared to pre-trained offline; the partial model update improves it by 12.34∼50.92%, which indicates a competitive accuracy compared to the entire model update. The results indicate that both update strategies show consistent trends for all the employed models over time.

Figure 4d and Figure 5d represent the accuracy of BERT-based architecture over time when each word embedding strategy (i.e., (1) Pre-trained offline, (2) Partial model update based on keyword-level word embedding, (3) Partial model update based on BERT Embedding) has been adopted on the CSI and disaster datasets, respectively. We note that both partial update models show a distinct performance improvement compared to the pre-trained offline model. The partial model update based on keyword-level word embedding, which is fully trained using the datasets defined for a target event, improves the classification accuracy by 21.21∼26.60% compared to the pre-trained offline model; that based on BERT embedding improves it by 12.08∼22.70%.

From the experimental results on both datasets, we point out the following three major observations. (1) The entire update strategy gradually increases the model accuracy for all the underlying deep learning-based models as time increases. This indicates that the accumulated data contribute to the improvement of the model performance. (2) The partial update strategy significantly improves the model accuracy of the pre-trained offline model and shows a competitive accuracy to the entire model update with a marginal model update overhead while maintaining the accuracy over time. This indicates that the partial update strategy can deal with the changes in the event effectively. (3) BERT is generally known to perform well by learning a large amount of data. However, despite fine-tuning, it does not perform well on the event-specific datasets targeted in this study compared to the other fully trained models using event-related datasets. To verify this, we compare BERT-based architecture using the original BERT embedding model with it using the event-specific word embedding for the other models. The results confirm that the model based on the original BERT embedding shows a classification accuracy of 0.8014 for the CSI dataset and 0.7833 for the disaster dataset, which are lower than 5.98% and 8.07% compared to the model based on the event-specific word embedding, respectively. This indicates that specific events have to be represented by a distinct set of keywords specifically related to those events, and so we need to build our own language models to represent each event.

#### 4.3.2. Model Learning Time

Table 4 and Table 5 represent the model learning time on the CSI and disaster datasets, respectively, for each time window of the following different update strategies: (1) no online update, (2) entire model update, and (3) partial model update. Here, we note that as more data are accumulated over time, the model learning time of the entire model update proportionally increases. In contrast, the partial update strategy consistently maintains the learning time as time varies on both datasets. We partially update the model by fine-tuning only the final dense layer to all the models in common. However, we observe that the model learning time significantly differs depending on the model. This stems from the inference complexity of each model because the inference from the fixed model is required for the input of the dense layer. In the case of BERT, we fine-tune only the dense layer through SparkNLP by using the learned transformer without fitting it. Accordingly, its learning time is comparatively short considering the massive-scale pre-trained model of BERT.

#### 4.3.3. Scalability on a Cluster

Table 6 shows the elapsed time for learning the classification model using 50,000 tweets as the number of worker nodes in the Spark cluster increases. Here, we use 10 epochs to train the Bi-LSTM model in Section 4.2 and assign one executor to each node consisting of five cores. The results indicate that the learning time of both entire and partial model updates effectively decreases as the number of worker nodes increases. Specifically, it decreases the learning time by 34.03% in the entire model update and by 45.21% in the partial model update, respectively, verifying the scalability of the proposed distributed learning pipeline. In particular, in the entire update model, this scalable architecture alleviates its learning overhead by scaling the distributed environments as we want. In the case of the model inference time as well, we can observe an explicit tendency where the inference time significantly decreases as the number of worker nodes increases. Compared to using a single worker node, a distributed configuration of using three worker nodes decreases the inference time by 57.41% in the entire model update and by 50.37% in the partial model update, respectively. Although it only requires a relatively very short time compared to the learning time, decreasing the inference time is quite important in real-time classification.

#### 4.3.4. Case Study

**Figure 6:**
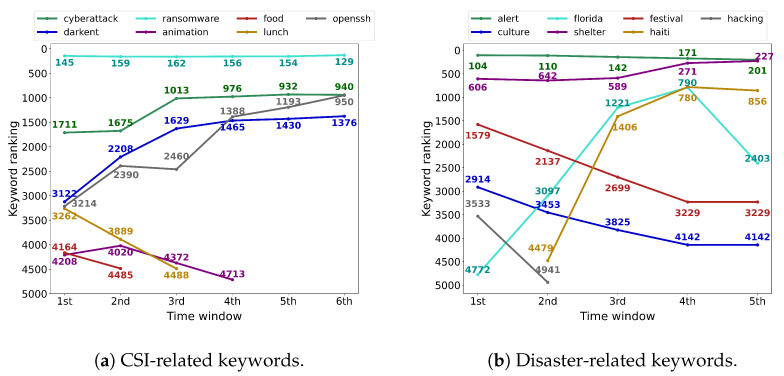
The change of keyword ranking over time.

Figure 6 shows the ranking changes of selected keywords based on the frequency over time. As explained in Section 3, we update the list of keyword tokens and their ranking in each time window. The result indicates that most of the relevant keywords extracted from the pre-trained offline model have been maintained or become risen in the top keyword sets, whereas general keywords that are less important to the target events are removed from the top keyword sets.

As shown in Figure 6a on the CSI dataset, the ranking of highly relevant keywords, such as ‘cyberattack’ and ‘ransomware’, has maintained over time. On the other hand, the ranking of keywords associated with a particular cyber-security event, such as ‘darknet’ and ‘openssh’, is rapidly increasing as the actual event occurs. In contrast, the ranking of the keywords that were included in the pre-trained offline model, but are not actually relevant to the target event continues to decrease over time, such as ‘animation’, ‘food’, and ‘lunch’.

A similar tendency was observed in Figure 6b on the disaster dataset. That is, the ranking of highly relevant keywords, such as ‘shelter’ and ‘alert’, has maintained in the ranking of keywords over time; the ranking of keywords associated with a particular disaster, such as ‘florida’ and ‘haiti’, rapidly increases. In contrast, the ranking of the keywords that are not actually relevant to the target event, such as ‘hacking’, ‘festival’, and ‘culture’, decreases over time. Here, we note that the degree of changes in the keyword ranking in the disaster dataset is relatively dynamic compared to the CSI dataset. This stems from the nature of the disaster events where completely different kinds of new events occur, in contrast to the cyber-security domain where the used terms are limited across the domain and domain-specific terms have been continuously used. This indicates that we need to effectively update the word embedding to reflect newly important keywords and eliminate less important keywords so that we can effectively track the event changes.

## 5. Conclusions and Future Work

In this study, we investigated the problem of dynamically updating the classification model that can adaptively respond to changes in real-time data streams in a distributed learning environment. We proposed two dynamic model update methods: (1) entire model update and (2) partial model update. The former updated the entire model using the accumulated datasets, maximizing the model accuracy with excessive learning overheads; the latter updated a part of the model using only a limited number of recent datasets, maximizing the learning efficiency while maintaining reasonable accuracy. A scalable architecture based on Apache Spark can effectively resolve the bottleneck that occurred during the learning process in the entire event stream classification pipeline. Through the extensive experiments using two real-world tweet datasets, we showed that the entire model update improved the classification accuracy by 28.96∼58.63% compared to the pre-trained offline model, while its learning overhead incrementally increases. On the other hand, the partial model update improves the accuracy by 12.34∼50.92%, while significantly reducing the learning time by 69.35% up to 93.95% compared to the entire model update strategy.

In this study, we focused on the dynamic model update in a distributed environment with the efficient learning methods to reflect new tweet streams to the model. However, as the orders of the event trends are not predictable in practice, the classification model not only requires learning new tasks but also needs to effectively maintain the learned representations of the previous tasks. While the existing model can be adapted to the current task by incrementally learning the current task based on the previously learned representations, they are prone to catastrophic forgetting, i.e., forgetting the previously learned representations [40]. In this study, we did not focus on maintaining the model performance for previous tasks, but they are required when the previous tasks are performed repeatedly. Therefore, as a further study, we plan to investigate continual learning algorithms based on a distributed environment to resolve those challenges. To achieve this, we will manage separate models for specific tasks, and each model would be updated differently according to the degree of event changes in data streams.

## Figures and Tables

**Table 1:** The elapsed time for ingesting, learning, and inferencing tweets.

Number of Tweets	Ingestion Time	Learning Time	Inference Time
50,000	266 s	1120 s	0.0671 s
100,000	523 s	2467 s	0.0784 s
150,000	751 s	4076 s	0.0854 s
200,000	1094 s	5604 s	0.1345 s

**Table 2:** Comparison between the previous studies and our model.

Papers	Streaming Classification	Distributed Learning	Dynamic Model Update	Model Learning Efficiency
[5]	O		O	
[7]	O			
[12]	O		O	
[14]	O	O		O
[20]		O		O
[23]	O	O	O	
[29]		O		
[30]	O	O		
[40]	O		O	
[48]			O	
[51]		O	O	
[52]	O		O	O
Our model	O	O	O	O

**Table 3:** The used twitter datasets.

Dataset Name	Total Data Size	Number of Time Windows	Target Written Duration (Years)
CSI Dataset	1,000,000 tweets	6	2007 ∼ 2015
Disaster Dataset	1,400,000 tweets	5	2007 ∼ 2015

**Table 4:** The learning time of the proposed strategies on the CSI dataset (seconds).

		1st Time Window	2nd Time Window	3rd Time Window	4th Time Window	5th Time Window	6th Time Window
CNN	Entire	489.35	530.83	790.23	1040.21	1250.54	1560.38
	Partial	130.13	143.62	131.99	156.23	139.37	147.01
RNN	Entire	300.1	440.63	532.44	640.34	784.78	838.81
	Partial	91.4	97.17	86.66	78.28	75.91	73.26
Bi-LSTM	Entire	1550.45	1750.27	2250.17	3240.83	4010.45	4630.2
	Partial	1430.59	1382.2	1357.06	1436.02	1402.12	1419.21
BERT	Partial	60.36	62.27	70.23	51.29	54.33	58.15

**Table 5:** The learning time of the proposed strategies on the disaster dataset (seconds).

		1st Time Window	2nd Time Window	3rd Time Window	4th Time Window	5th Time Window
CNN	Entire	210.16	745.88	1140.34	1754.71	2165.9
	Partial	138.45	131.62	127.17	134.8	131.01
RNN	Entire	173.3	517.62	820.29	1209.09	1876.43
	Partial	116.17	112.53	109.8	128.41	115.69
Bi-LSTM	Entire	1840.23	4073.32	7580.61	8073.73	12587.11
	Partial	1770.3	1520.13	1646.1	1602.36	1457.14
BERT	Partial	159.66	127.52	133.68	149.84	130.62

**Table 6:** The elapsed time for training model by the number of worker nodes using proposed strategies (seconds).

Update Strategy	Number of Worker Nodes	Learning Time	Inference Time
Entire Model Update	1	1834.23	0.1599
	2	1587.05	0.0967
	3	1210.04	0.0681
Partial Model Update	1	1668.87	0.1362
	2	1424.88	0.0857
	3	914.30	0.0676

## Data Availability

The data presented in this study are available on request from the corresponding author. The data are not publicly available due to privacy.

## Abbreviations

The following abbreviations are used in this manuscript:

CNN	Convolutional Neural Network
RNN	Recurrent Neural Network
LSTM	Long Short-Term Memory
NLP	Natural Language Processing
BERT	Bidirectional Encoder Representations from Transformers
CSI	Cyber-Security Intelligence
Bi-LSTM	Bidirectional Long Short Term Memory
